# Prognostic Value of Functional Assessment of Cancer Therapy-General (FACT-G) in Advanced Non-Small-Cell Lung Cancer Treated with Korean Medicine

**DOI:** 10.1155/2020/2845401

**Published:** 2020-01-20

**Authors:** Hyeonjin Jeon, Wankyu Eo, Bumsang Shim, Sehyun Kim, Sookyung Lee

**Affiliations:** ^1^Department of Clinical Korean Medicine, Graduate School, Kyung Hee University, Seoul, Republic of Korea; ^2^Department of Medical Oncology & Hematology, College of Medicine, Kyung Hee University, Seoul, Republic of Korea; ^3^Department of Pathology, College of Korean Medicine, Kyung Hee University, Seoul, Republic of Korea; ^4^Graduate School, Dankook University, Yongin, Republic of Korea; ^5^Department of Clinical Oncology, College of Korean Medicine, Kyung Hee University, Seoul, Republic of Korea

## Abstract

**Objectives:**

The impact of health-related quality of life (HRQoL) on survival has been investigated in patients with various cancers. Here, we evaluated the prognostic value of HRQoL using the Functional Assessment of Cancer Therapy-General (FACT-G) in advanced non-small-cell lung cancer (NSCLC) patients treated with Korean medicine.

**Methods:**

A retrospective review of medical records and FACT-G scores of patients with advanced NSCLC who received treatment with Korean medicine was conducted. The reliability of the FACT-G was determined using Cronbach's alpha and calculating floor-and-ceiling effects. Correlations between FACT-G scores were estimated using Pearson's correlation analysis. Overall survival was calculated using the Kaplan–Meier method, and the prognostic impact of FACT-G scores and patients' characteristics was evaluated with Cox proportional hazards regression.

**Results:**

Of the 165 enrolled patients, 115 (70%) had extrathoracic metastasis and 139 (84%) had undergone prior anticancer treatment. The median overall survival was 10.1 months. The mean FACT-G score was 65.0, and Cronbach's alpha for the FACT-G was 0.917. Age ≥65 years, male sex, smoking history, squamous-cell carcinoma, Eastern Cooperative Oncology Group Performance Status (ECOG-PS) ≥2, and presence of extrathoracic metastasis were associated with an increased risk of mortality. High FACT-G total scores, physical well-being (PWB), emotional well-being, and functional well-being were associated with prolonged survival. After adjusting for age, sex, smoking history, ECOG-PS, histological type, and presence of extrathoracic metastasis, a high FACT-G total score (hazard ratio (HR): 0.99, *p*=0.032) and high PWB score (HR: 0.94, *p* < 0.001) were associated with prolonged survival as independent prognostic factors in patients with advanced NSCLC.

**Conclusion:**

The FACT-G total score and PWB score as HRQoL measurements were significant prognostic factors for survival in advanced NSCLC patients treated with Korean medicine. This finding implies that the FACT-G can be used in clinical practice as a predictor of survival in patients with advanced NSCLC.

## 1. Introduction

Lung cancer is the leading cause of cancer deaths worldwide, with an estimate of 1.76 million deaths in 2018 [1]. In particular, non-small-cell lung cancer (NSCLC) comprises about 84% [2] of all lung cancer cases, 55% of which are reported to be diagnosed at the metastatic stage [3]. Due to the limited treatment options and relatively short survival, patients with advanced NSCLC should be treated effectively at every decision point. Health-related quality of life (HRQoL) in clinical practice is an important factor used to decide the optimal care for patients, considering benefits and risks of treatment modalities and anticancer therapies. Regarding the outcomes of cancer treatment, the American Society of Clinical Oncology suggested that HRQoL, as a patient-reported outcome, should receive higher priority than tumor response, which has been commonly evaluated as an endpoint of tumor-related outcomes [4]. Recently, HRQoL reflecting various aspects of the patient's condition has gained importance as an endpoint to determine the effectiveness of anticancer therapy in clinical trials [5]. Indeed, several studies showed a positive relationship between HRQoL and survival [6–9]. In the clinical practice of advanced NSCLC, an important issue is HRQoL management, which deteriorates as disease progresses. In some cases, physical discomfort may cause psychological stress that leads to limitations in social activities among these patients [10–13]. Therefore, HRQoL is commonly used as one of the main endpoints in clinical trials to evaluate the effectiveness of anticancer therapies for advanced NSCLC [14, 15].

To identify the association between HRQoL and survival, several HRQoL measurement instruments have been extensively investigated with respect to their prognostic impact on survival in patients with cancer [6–9]. One of the global HRQoL instruments, the European Organization for Research and Treatment of Cancer Quality of Life Questionnaire-Core 30 (EORTC QLQ-C30), has been reported to have a better prognostic capability than performance status for survival of patients with lung cancer [16], colorectal cancer [17], and aggressive lymphoma [18]. In particular, the global QoL status of patients with aggressive lymphoma shows a stronger prognostic impact on survival than the Ann Arbor Staging System of lymphoma [18]. In addition, the EORTC QLQ-C30 has showed a significant association with survival in patients with NSCLC [19–22] and advanced NSCLC [16, 23].

However, few studies using the Functional Assessment of Cancer Therapy-General (FACT-G) have been performed in patients with advanced NSCLC. While the EORTC QLQ-C30 assesses global QoL as a single item, the FACT-G estimates HRQoL using four different domains (physical, social/family, functional, and emotional well-being), and global HRQoL is represented as a total score that is the sum of all domains [24]. The assessment of global HRQoL using different domains evaluates HRQoL using balanced aspects of life and assesses it through independent subdomains. Two studies have reported an association between FACT-G and survival in patients with lung cancer, including small-cell lung cancer (SCLC) [25, 26]. One study was conducted on a total of 42 small groups [25], and the other focused on patients with various stages of lung cancer without considering their histological subtype [26]. However, the overall survival and QoL of patients with lung cancer considerably differ depending on the cancer stage, especially between early and advanced stages, as well as on the histological type of tumor, SCLC or NSCLC [3, 27]. Therefore, this study aimed to investigate the prognostic value of the FACT-G in the survival of advanced NSCLC patients treated with Korean medicine (KM). After verifying the reliability of the FACT-G scores, the prognostic impact of the FACT-G total score and its subdomain scores was evaluated controlling for other potential prognostic factors. The findings of this study could provide evidence on the use of the FACT-G for predicting the outcome of patients with advanced NSCLC.

## 2. Materials and Methods

In this study, we retrospectively reviewed the health records and FACT-G scores of patients with advanced NSCLC, under the approval of the Institutional Review Board of the Kyung Hee University Hospital at Gangdong.

### 2.1. Patients

This study included patients who visited our cancer center to treat or manage their disease using KM. We enrolled patients with lung cancer who had been managed from June 2006 to December 2015. The inclusion criteria were as follows: (1) histologically confirmed NSCLC; (2) diagnosed stage IIIB or IV disease at the initial visit, according to the American Joint Committee on Cancer/Union for International Cancer Control (AJCC/UICC) Staging Manual, 7th edition [28]; and (3) FACT-G questionnaire completed within 1 month of the first visit day. We excluded patients whose FACT-G questionnaires were unsuitable to calculate scores according to the FACT-G scoring guidelines.

### 2.2. FACT-G

We used the FACT-G Korean version (version 4), which has been validated in Korean patients with cancer, under the permission of the Functional Assessment of Chronic Illness Therapy Organization [29–31].

The FACT-G consists of 27 items grouped into four general subdomains: physical well-being (PWB; 7 items, score range: 0–28), social/family well-being (SFWB; 7 items, score range: 0–28), emotional well-being (EWB; 6 items, score range: 0–24), and functional well-being (FWB; 7 items, score range: 0–28). The responses to each item are rated on a 5-point Likert scale ranging from 0, “not at all,” to 4, “very much,” depending on the HRQoL experienced by the patients within the previous 7 days. To calculate the FACT-G score, more than 50% of the items in each subdomain and 80% of the total items must be answered. The score was calculated according to the FACT-G scoring guidelines, and the scores of reverse items were subtracted from 4. If there were missing items, the score was calculated using a proportional distribution method, in which the sum of the scores of the answered items was multiplied by the total number of items in the subdomain and divided by the number of answered items. The total FACT-G score consists of the sum of the four subdomains, ranging from 0 to 108. A higher score indicates a better HRQoL.

For each patient, the survey was performed by medical staff who were not in charge of the patient, and each item of the FACT-G questionnaire was answered by the patients themselves.

The reliability of the FACT-G was statistically assessed using Cronbach's alpha. In the statistical analysis, FACT-G scores were compared and analyzed as continuous variables because an optimal cut-off value for FACT-G scores has not been established.

### 2.3. Variables

Clinical factors such as age, sex, performance status, smoking history, histological type, TNM staging, and presence of extrathoracic metastasis were investigated. The history of anticancer treatment and the combination of conventional anticancer therapy were also reviewed to investigate their influence on survival. Candidate clinical factors were analyzed after dichotomization, according to reference values of clinical relevance. Age was dichotomized as elderly and young patients, with the age of 65 years corresponding to elderly. Smoking history was dichotomized as never smoker versus ex-smoker or current smoker. Performance status was assessed with the Eastern Cooperative Oncology Group Performance Status (ECOG-PS) score and was classified as 0-1 or ≥2. Tumor histological type was classified as squamous-cell carcinoma and non-squamous-cell carcinoma, and patients with pathologically confirmed unspecified NSCLC were included in the non-squamous-cell carcinoma group.

### 2.4. Treatment

Enrolled patients were treated with KM, including herbal medicine and acupuncture. The main antitumor agent was the *Rhus verniciflua* Stokes (RVS) extract, which has been reported to have antitumor activity, such as induction of apoptosis, inhibition of proliferation and migration, and inhibition of angiogenesis [32–34]. The aqueous RVS extract was concentrated and lyophilized; the toxic allergen urushiol was removed, and the quality of the extract's main components was controlled. Five hundred milligrams of the lyophilized RVS extract were administered in the form of capsules, three times a day. In case of requiring management of the patient's symptoms or disease status, acupuncture and herbal medications were administered, according to KM guidelines. The combination of conventional anticancer therapy, such as chemotherapy or radiotherapy, was also investigated.

### 2.5. Statistical Analysis

Demographic and clinical characteristics of participants were analyzed with descriptive statistics and frequency analysis. FACT-G scores were analyzed using mean, standard deviation, and range. For the validation of quality, Cronbach's alpha analyzed the reliability of internal consistency of the FACT-G and the floor-and-ceiling effects examined sensitivity and responsiveness.

The overall survival time was defined as the period from the date of FACT-G until the date of death from any cause. Patients who were alive at the end of the study or those whose survival could not be confirmed were censored at the date of the survival investigation day (September 12, 2017). Survival curves were estimated with the Kaplan–Meier method.

The prognostic significance of FACT-G scores was analyzed using Cox proportional hazards regression. Univariate analysis was performed with potential prognostic factors including demographic and clinical characteristics. Multivariate analysis was conducted using the full model method after adjusting for candidate prognostic factors that showed a significant influence on survival in univariate analyses. Multivariate modeling was performed with three different models considering the multicollinearity that resulted from the correlation between the FACT-G total score and the score of each subdomain [35]. Model 1 was analyzed with demographic and clinical factors, including age, sex, smoking history, ECOG-PS, histological type, and presence of extrathoracic metastasis; model 2 was analyzed by adding the FACT-G total score into model 1; model 3 was analyzed by adding the scores of each FACT-G subdomain to model 1, instead of the FACT-G total score.

The correlations between FACT-G scores were analyzed using Pearson's correlation analysis, and the multicollinearity of variables in multivariate analysis was examined using the value of variance inflation factors.

Statistical analysis was conducted with SPSS, version 18.0 (SPSS Inc., Chicago, IL, USA), and probability values less than 0.05 were considered statistically significant.

## 3. Results

**Figure 1:**
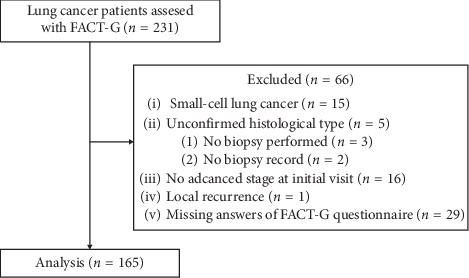
Flow diagram of patient enrollment.

Of the 231 lung cancer patients who underwent KM treatment and had available data of FACT-G scores, 66 were excluded; thus, 165 patients were included in the analysis (Figure 1). Demographic and clinical characteristics of the patients are shown in Table 1. Fifty-nine patients (36%) were elderly patients (aged 65 years and above), and 75 patients (45%) had ECOG-PS ≥2. The main histological tumor type was adenocarcinoma (*N* = 110; 67%). Most patients (*N* = 154; 93%) presented with stage IV NSCLC, and 115 patients (70%) had extrathoracic metastasis. The majority of patients (*N* = 139; 84%) had undergone prior anticancer therapy, and 69 patients (42%) underwent KM therapy concurrently with chemotherapy or radiotherapy. The median duration from the date of initial diagnosis to the date of the FACT-G was 9.4 (range: 0.3–173) months, and the median duration from the date of metastasis diagnosis to the date of the FACT-G was 6.3 (range: 0–133) months.

The FACT-G scores of all enrolled patients are shown in Table 2. Cronbach's alpha of the FACT-G total score was 0.917, showing acceptable reliability, and the percentage of floor-and-ceiling effects was less than 5% in each subdomain, presenting good sensitivity and responsiveness. The FACT-G total score and subdomain scores showed strong associations; lower correlations were obtained among subdomain scores (Table 2).

The median overall survival time was 10.1 months (95% confidence interval (CI): 8.3–11.7). There was no difference in survival according to the combination of conventional anticancer therapy (*p*=0.781). In univariate analysis, age, sex, smoking history, ECOG-PS, histological tumor type, presence of extrathoracic metastasis, FACT-G total score, PWB score, EWB score, and FWB score were significant prognostic factors for survival (Table 3). Among clinical variables, TNM staging, history of anticancer therapy, and combination of conventional anticancer therapy did not affect overall survival as prognostic factors (Table 3). In multivariate analysis, ECOG-PS and presence of extrathoracic metastasis were identified as significant prognostic factors in all models, and the FACT-G total score (model 2) and the PWB score (model 3) were identified as significant prognostic factors for survival in patients with advanced NSCLC (Table 3).

## 4. Discussion

In this study, the quality of FACT-G as a suitable instrument for patient-reported HRQoL was proven by its high Cronbach's alpha value and low percentage of floor-and-ceiling effects. The results of correlations analysis between the FACT-G total score and subdomain scores indicated that each subdomain was associated with the global HRQoL, reflecting the characteristics of different aspects of the HRQoL.

Based on the results of this study, the FACT-G total score and the PWB score were independent prognostic factors for survival in patients with advanced NSCLC, which implies that high FACT-G total scores and high PWB scores were associated with prolonged survival and lower risk of mortality. Furthermore, the prognostic value of the FACT-G total score and the PWB score was comparable with that of the ECOG-PS or the presence of extrathoracic metastasis, which are well-known prognostic factors for survival in patients with advanced NSCLC.

The finding that the total FACT-G score predicts survival implies that the patients' self-reported QoL can serve as a precise overall indicator of the symptom burden to the medical staff and that HRQoL measurements can be applied as important instruments to communicate with patients in clinical practice. This crucial prognostic information provided by the FACT-G total score as a measurement of the global HRQoL shows similar results than those obtained with the EORTC QLQ-C30 in the management of patients with advanced NSCLC [16, 23]. The survival predictive value of the PWB score is deemed reasonable and appropriate, considering the fact that the PWB score directly reflects the patients' physical condition.

Previous studies evaluating the prognostic impact of the FACT-G on survival of patients with lung cancer have shown discrepancies with our results. In a study that enrolled patients regardless of their disease stage and histological subtype, the prognostic impact of the FACT-G total score was not significant, but only that of the PWB score was significant [26]. Other studies performed with patients in stages III and IV have shown conflicting results [25, 36]. These conflicting results on the utility of the FACT-G total score may be explained by the heterogeneity of the enrolled patients in terms of their disease stage and subtype of lung cancer, which influence survival. The diverse stages of cancer, from early to advanced, can reduce the prognostic impact of the FACT-G on survival because of differences in disease courses and treatment strategies [37].

In addition, the proportion of patients with SCLC in the study population might influence survival and QoL due to the distinctive characteristics of SCLC such as rapid growth, early metastasis, high response to initial treatment, and frequent relapse from cytotoxic chemotherapy, which result in limitations in the predictive value of the FACT-G on survival [38]. As reflected in the different prognoses observed according to SCLC and NSCLC subtypes, Dharma-Wardene et al. showed a significant higher hazard ratio (HR: 5.88, *p*=0.006) in patients with SCLC compared with patients with NSCLC [25]. Regarding HRQoL and depression in patients with lung cancer, the latter was more frequently reported in patients with SCLC than in patients with NSCLC [39]. Therefore, considering the outcomes of previous studies, the impact of HRQoL on survival in patients with lung cancer should be evaluated distinguishing the different subtypes of SCLC and NSCLC.

Among the subscales of the FACT-G, PWB was identified as an independent prognostic factor for survival. Considering the importance of the physical condition in the HRQoL, PWB was a confirmed decisive factor that influenced and predicted survival independently. Based on this result, the management of physical status in clinical practice should be emphasized in order to prolong survival time in patients with advanced NSCLC. Considering the prognostic significance of the FACT-G total score and the PWB score in patients with advanced NSCLC, the FACT-G can be a useful guide to choose optimal treatment options and to manage patients' symptoms in clinical practice.

The findings of the present study imply that management of patients based on patient-reported outcomes (PROs) in routine cancer care may improve the quality of life and prolong survival, consistent with previous studies [40, 41].

This study was performed in a clinical cancer center based on KM. Cancer treatment with KM is centered on treating patients rather than treating tumors and focuses on alleviating symptoms and improving the quality of life from a holistic perspective. In contrast to conventional anticancer therapy, the use of herbs and acupuncture as treatment modalities is relatively nontoxic, and hence, adverse events are rare [42]. Besides, the main anticancer agent used in this study was administered to patients after depleting the toxic allergen urushiol. These characteristics of cancer treatment with KM may have affected the outcome, increasing the survival of patients with good HRQoL.

As this study evaluated the actual situation in clinical practice, the FACT-G was performed at the initiation of KM treatment. Thus, most patients had received prior conventional anticancer therapy and had refractory or relapsed advanced NSCLC. Some of them received concurrent conventional anticancer therapy. However, the history of prior anticancer therapy and combination of conventional anticancer therapy had no influence on survival, while the total FACT-G score and its subdomain scores showed a significant influence on survival as prognostic factors.

Based on the result of this study, patients with good HRQoL might be expected to have prolonged survival. Considering the result of the present study and the previous study using the EORTC QLQ-C30 [16, 23], maintaining a high HRQoL might be an effective treatment as reducing tumor burden for the management of patients with advanced NSCLC. Until recently, the evaluation of the effectiveness of anticancer therapy had focused on tumor size reduction [43]; however, an increasing number of studies prove that quality of life affects the survival of patients with cancer. Patient-centered care can be universalized, and a patient-centered anticancer therapy will probably play a more active role in clinical practice in the years to come.

Besides the FACT-G total score and the PWB score, a high ECOG-PS score and the presence of extrathoracic metastasis were also independent prognostic factors for survival, demonstrating the high risk of mortality in patients with advanced NSCLC. As a well-known predictor of survival, ECOG-PS represents the physical status of a patient graded by a physician, and the presence of extrathoracic metastasis represents the anatomical extent of disease status as a disease-related factor.

As a retrospective observational study, this study has limitations to be considered. First, this study was performed with restricted access to some clinical characteristics of the patients' health information such as changes of body weight, dietary assessment, and various socioeconomic factors, which also affect QoL. Second, in this study, the FACT-G questionnaire was assessed at the initial visit to our clinic of KM treatment. Thus, although some patients assessed the FACT-G immediately after diagnosis, most patients were evaluated after diagnosis of relapsed or refractory advanced NSCLC, having had previous experiences with conventional anticancer treatment. Third, this study analyzed the FACT-G scores as continuous variables in order to confirm the value of original data of FACT-G scores in the absence of established cut-off values, whereas other candidate clinical factors were analyzed with dichotomized variables. This may lead to limitations in the interpretation of the results and their clinical implications. Fourth, as this was a retrospective observational study based on real-world clinical data, some patients received conventional anticancer therapy during KM treatment. To confirm these results and to expand their application in the clinical practice, a well-designed prospective study is necessary to provide strong evidence to the prognostic value of FACT-G on survival. In addition, if future studies are performed with multiple measurements of FACT-G scores during the treatment course or disease progression, the information on the change in HRQoL will provide valuable perspectives for cancer treatment, and this information could be applied for patient management in clinical practice.

## 5. Conclusions

Despite these limitations, this study demonstrated the independent prognostic value of the FACT-G as a global HRQoL measurement of patient survival, which is comparable to the ECOG-PS in advanced NSCLC patients treated with KM. This study implies that the management of HRQoL may affect the length of overall survival in real-world clinical practice.

## Figures and Tables

**Table 1:** Demographic and clinical characteristics of patients.

	*N*	%
Sex		
Men	93	56
Women	72	44
Age (years)		
<65	106	64
≥65	59	36
Smoking		
Never smoker	81	49
Ex-smoker or current smoker	84	51
ECOG-PS		
0-1	90	55
≥2	75	45
Histological type		
Adenocarcinoma	110	67
Squamous-cell carcinoma	29	18
Others	26	16
Stage		
IIIB	11	7
IV	154	93
Extrathoracic metastasis		
No	50	30
Yes	115	70
History of anticancer treatment		
No	26	16
Yes	139	84
Treatment		
KM monotherapy	96	58
KM with conventional therapy	69	42

Abbreviations: ECOG, Eastern Cooperative Oncology Group; PS, performance status; KM, Korean medicine.

**Table 2:** Distribution, reliability, and correlation coefficients of FACT-G scores.

	FACT-G	PWB	SFWB	EWB	FWB
Mean ± SD	65.0 ± 17.7	18.2 ± 6.4	18.0 ± 6.1	14.5 ± 5.5	14.4 ± 6.9
Median	64	19	18.7	15	14
Possible range	0–108	0–28	0–28	0–24	0–28
Observed range	19.4–107	3–28	0–28	0–24	0–28
Floor effects (%)	0	0	1 (1%)	2 (1%)	1 (1%)
Ceiling effects (%)	0	5 (3%)	8 (5%)	4 (2%)	3 (2%)
Cronbach's alpha	0.917	0.850	0.828	0.809	0.860
Correlation coefficients					
FACT-G		0.668^*∗∗*^	0.583^*∗∗*^	0.709^*∗∗*^	0.861^*∗∗*^
PWB			−0.013	0.398^*∗∗*^	0.474^*∗∗*^
SFWB				0.214^*∗∗*^	0.454^*∗∗*^
EWB					0.466^*∗∗*^

^*∗∗*^ *p* < 0.001 . Abbreviations: EWB, emotional well-being; FACT-G, Functional Assessment Cancer Therapy-General; FWB, functional well-being; PWB, physical well-being; SD, standard deviation; SFWB, social/family well-being.

**Table 3:** Univariate and multivariate analysis for overall survival.

	Univariate analysis		Multivariate analysis					
			Model 1		Model 2		Model 3	
Variables	HR (95% CI)	*p* value	HR (95% CI)	*p* value	HR (95% CI)	*p* value	HR (95% CI)	*p* value
Sex								
Men	1.79 (1.28–2.51)	<0.001	1.51 (0.90–2.53)	0.116	1.76 (1.03–3.02)	0.04	1.77 (1.01–3.07)	0.045
Women	1.00		1.00		1.00		1.00	
Age (years)								
<65	1.00		1.00		1.00		1.00	
≥65	1.66 (1.18–2.33)	0.003	1.23 (0.85–1.77)	0.266	1.22 (0.85–1.75)	0.290	1.48 (1.01–2.17)	0.046
Smoking history								
Never smoker	1.00		1.00		1.00		1.00	
Ex-smoker or current smoker	1.76 (1.26–2.44)	<0.001	1.03 (0.61–1.73)	0.912	0.99 (0.59–1.67)	0.973	1.10 (0.65–1.86)	0.723
ECOG-PS								
0-1	1.00		1.00		1.00		1.00	
≥2	3.01 (2.14–4.23)	<0.001	2.51 (1.71–3.67)	<0.001	2.18 (1.47–3.25)	<0.001	1.73 (1.14–2.63)	0.011
Histological type								
Non-squamous-cell carcinoma	1.00		1.00		1.00		1.00	
SCC	2.20 (1.44–3.37)	<0.001	1.30 (0.81–2.09)	0.284	1.22 (0.75–1.97)	0.421	1.21 (0.75–1.94)	0.442
Extrathoracic metastasis								
No	1.00		1.00		1.00		1.00	
Yes	1.69 (1.17–2.43)	0.005	1.90 (1.31–2.77)	<0.001	1.78 (1.22–2.61)	0.003	1.71 (1.17–2.51)	0.006
FACT-G total scores	0.98 (0.97–0.99)	<0.001			0.99 (0.98–1.00)	0.032		
PWB	0.93 (0.91–0.95)	<0.001					0.94 (0.91–0.97)	<0.001
SFWB	1.00 (0.98–1.03)	0.715					1.01 (0.97–1.04)	0.716
EWB	0.96 (0.93–0.99)	0.005					0.98 (0.95–1.02)	0.416
FWB	0.97 (0.95–0.99)	0.005					1.01 (0.97–1.04)	0.634
Stage								
IIIB	1.00							
IV	1.05 (0.53–2.06)	0.890						
History of anticancer treatment								
No	1							
Yes	0.70 (0.45–1.08)	0.103						
Treatment								
KM monotherapy	1							
KM with conventional therapy	1.05 (0.75–1.46)	0.780						

Abbreviations: CI, confidence interval; ECOG, Eastern Cooperative Oncology Group; EWB, emotional well-being; FACT-G, Functional Assessment Cancer Therapy-General; FWB, functional well-being; HR, hazard ratio; PS, performance status; PWB, physical well-being; SCC, squamous-cell carcinoma; SFWB, social/family well-being.

## Data Availability

The data used to support the findings of this study are available from the corresponding author upon request.
